# From pilots to platforms: making digital health work for chronic disease management

**DOI:** 10.3389/fpubh.2026.1780035

**Published:** 2026-05-22

**Authors:** Do-Youn Lee, Dinesh Puri, Amit Kumar Gupta, Preeti Dnyandeo Sonje, Sorabh Lakhanpal, Sujeet Kumar Singh, Sanjay Kumar, Prashant Ramdas Kokiwar

**Affiliations:** 1 College of General Education, Kookmin University, Seoul, Republic of Korea; 2 Faculty of Pharmaceutical Sciences, Graphic Era Hill University, Dehradun, India; 3 Centre for Promotion of Research, Graphic Era Deemed University, Dehradun, India; 4 Department of Radiology, School of Medical Sciences and Research, Sharda University, Greater Noida, India; 5 Department of Anatomy, Dr. D. Y. Patil Medical College, Hospital and Research Centre, Dr. D. Y. Patil Vidyapeeth (Deemed-to-be-University), Pune, Maharashtra, India; 6 School of Pharmaceutical Sciences, Lovely Professional University, Phagwara, India; 7 Department of Biotechnology, Noida Institute of Engineering and Technology (Pharmacy Institute), Greater Noida, India; 8 Centre for Research Impact and Outcome, Chitkara College of Pharmacy, Chitkara University, Rajpura, Punjab, India; 9 Department of Community Medicine, Malla Reddy Institute of Medical Sciences, Malla Reddy Vishwavidyapeeth, Hyderabad, Telangana, India

**Keywords:** chronic disease management, digital divide, digital health, implementation science, learning health systems, patient-reported outcomes, telehealth

## Abstract

Digital health technologies are increasingly used in chronic disease management, yet the literature still shows a persistent gap between promising pilot projects and durable health-system value. This perspective argues that the central challenge is not a shortage of digital innovation, but inadequate care-grade integration: the extent to which digital tools are embedded within clinical workflows, workforce roles, reimbursement arrangements, interoperability, data governance, and patient support. Drawing on recent reviews, implementation studies, qualitative syntheses, and value-framework papers across chronic disease settings, we examine why uptake remains uneven and why apparently successful tools often fail to scale. Three themes recur. First, evaluations still overemphasize acceptability and adoption relative to clinical, service, and equity outcomes. Second, sustained use depends on workflow fit, burden, trust, and patient preferences rather than usability alone. Third, equity and governance are often treated as downstream considerations instead of core design constraints. We propose a care-grade roadmap built on operational co-design, theory-informed implementation, equity-first delivery, multidimensional value assessment, and learning health-system feedback loops. Digital chronic care is more likely to deliver durable benefit when technologies are designed, evaluated, and funded as components of service redesign rather than as stand-alone tools.

## Introduction

Chronic diseases create sustained demand for care coordination, self-management support, long-term monitoring, and continuity across care settings. Digital health technologies (DHTs) including telehealth, remote monitoring, digital self-management tools, patient portals, and data-enabled care platforms are increasingly positioned as part of the response because they can extend care beyond the clinic, reduce travel burden, and support more continuous feedback between patients and care teams (1, 2).

At the same time, the evidence does not support a simple innovation narrative. Recent syntheses show rapid expansion in digital interventions for chronic disease management, but the benefits remain uneven across outcomes, settings, and populations (3, 4). Clinical gains are often mixed, while behavioral and service-delivery outcomes appear more favorable; moreover, implementation quality, contextual fit, and sustainability are frequently under-described (3, 4). This makes it difficult to distinguish between tools that are genuinely ready for routine care and tools that perform well only under pilot conditions.

This perspective argues that the core problem is inadequate care-grade integration. By care-grade integration, we mean the embedding of a digital tool within routine care pathways, staffing models, implementation strategies, reimbursement logic, interoperability arrangements, governance safeguards, and patient support. A digital tool may be technically functional and even acceptable to users, yet still fail to improve chronic care if it increases workload, fragments accountability, excludes digitally marginalized groups, or lacks a convincing value proposition for health systems.

## Scope and framing of the evidence

This article is a perspective, not a systematic review, and it does not report new empirical data. The purpose is interpretive: to explain why digital health in chronic disease management often underperforms at scale and to identify practical directions for more coherent evaluation and implementation.

The evidence included here was selected purposively to answer four questions: (1) what broad implementation patterns are visible across chronic disease DHTs; (2) what explains uneven patient and clinician uptake; (3) how do equity, governance, and digital literacy shape real-world value; and (4) what kinds of evaluation better support scale-up and sustainability. Priority was given to recent scoping reviews, qualitative syntheses, implementation studies, and framework papers relevant to chronic disease management, while a small number of foundational implementation references were retained to clarify concepts such as implementation outcomes and scale-up (3–13).

Examples from chronic obstructive pulmonary disease (COPD), chronic kidney disease (CKD), telehealth in rural settings, and low-resource health systems are used here not to imply disease-specific superiority, but because they illuminate recurring issues in workflow fit, burden, equity, governance, and value assessment. Table 1 summarizes the logic used to organize the evidence base.

**Table 1:** Analytic domains and rationale for the evidence included in this perspective.

Analytic domain	Why it was included	Evidence emphasized	Representative examples
Cross-condition implementation patterns	To identify recurring strengths and gaps across chronic disease DHTs rather than focusing on a single disease area.	Recent scoping and review-level evidence (3, 4).	Implementation gap; mixed outcome profile.
Patient experience and sustained use	To explain why apparently useful tools are not always adopted or retained over time.	Qualitative syntheses, preference studies, and mixed-methods work (5, 10, 16–18).	Burden, trust, personalization, perceived value.
Clinician workflow integration	To assess whether DHTs fit routine primary and specialty care work rather than pilot workflows.	Rapid reviews and implementation-oriented studies (6, 11, 12).	Workload, accountability, data quality, sustainability.
Equity and digital inclusion	To test whether scale-up may widen disparities when access, literacy, or infrastructure are weak.	Telehealth access studies, health-literacy work, and low-resource implementation studies (9, 13, 19, 20).	Broadband, affordability, digital literacy, community context.
Value and governance	To clarify what should count as benefit when judging readiness for scale.	Multi-stakeholder value frameworks and health-system learning approaches (7, 8, 23).	Clinical value, service value, data rights, governance.

## From promising tools to uneven routine care

The strongest cross-cutting message from the chronic disease literature is that digital health is no longer marginal, but neither is it routinely care-grade. A scoping review of 252 studies published between 2015 and 2023 found extensive activity across telehealth, mobile health, and remote monitoring, yet only a small minority of studies explicitly used implementation science frameworks and most concentrated on early outcomes such as acceptability, feasibility, and adoption (3). A complementary review focused on non-communicable disease management in primary care settings in low- and middle-income countries likewise found that digital interventions were associated with more consistent gains in behavioral and service-delivery outcomes than in clinical endpoints, and that uptake depended heavily on contextual and system factors (4).

Condition-specific studies reinforce this mixed picture. In COPD, digital interventions such as telehealth, apps, wearable monitoring, and telerehabilitation may improve access and support monitoring, but the evidence remains heterogeneous and integration into long-term care pathways is incomplete (1, 14, 15). In CKD, patient preferences for wearable appearance, format, and communication style demonstrate that design details influence willingness to engage (16), while mixed-methods work on digital self-management shows that patients may value educational content more than the tracking functions developers often foreground (10). Together, these findings suggest that the problem is not simply whether DHTs can work, but under what service conditions, for whom, and with what kind of support.

## Why uptake and scale remain limited

Uptake in chronic disease management should not be treated as a one-time yes-or-no decision. Typology work suggests that many people with chronic disease remain relatively “hypoconnected,” while only a minority use multiple health technologies regularly (17). A similar clustering approach in multiple sclerosis found that technology enthusiasm is unevenly distributed and that many people use digital tools mainly for non-health purposes (18). These patterns matter because they show that non-use is not merely resistance; it often reflects a mismatch between the design of the intervention and the realities of everyday illness management.

Qualitative syntheses add explanatory depth. Across chronic disease studies, patients repeatedly describe a balance between reassurance and burden: DHTs can enhance support, access, and perceived control, but they can also impose data-entry work, technical friction, and ambiguity about whether the effort is worth it (5). Clinicians report a parallel set of concerns around workflow disruption, added documentation, questionable data quality, training needs, liability, and uncertainty over how digital inputs should change decision-making (6).

These findings align with foundational implementation theory. Proctor and colleagues distinguished implementation outcomes such as acceptability, adoption, and feasibility from service outcomes and clinical outcomes, making clear that success in one domain does not guarantee success in another (11). The NASSS framework similarly emphasizes that nonadoption, abandonment, and failure to scale are often produced by complexity across the condition, technology, value proposition, adopter system, organization, wider system, and adaptation over time (12). In other words, uptake is an output of design, workflow, and organizational alignment not a problem that can be solved by better interfaces alone.

## Equity, digital literacy, and governance as design constraints

A second recurring problem is that equity is often acknowledged rhetorically but operationalized weakly. County-level evidence from rural Florida showed that higher digital divide scores were associated with lower telehealth use and poorer health outcome rankings, indicating that digital availability does not translate automatically into meaningful access (19). Work focused on low-income African American and Black men similarly argues that digital health literacy must be addressed directly within chronic disease self-management programs if digital delivery is not to amplify existing disparities (20). In low-resource settings, barriers such as poor access to devices, weak connectivity, low technical confidence, and negative prior experiences further shape whether DHTs can be used at all (9).

The implication is that equity cannot be left to *post hoc* subgroup analysis. Richardson and colleagues’ framework for digital health equity is useful here because it extends attention beyond individual skills to the digital environment at interpersonal, community, and societal levels (13). For chronic disease management, this means that low-bandwidth functionality, device affordability, language and literacy sensitivity, local care navigation, and trust in data governance are not optional enhancements; they are prerequisites for population-scale relevance.

Governance is part of this same problem. Chronic disease platforms increasingly depend on patient-generated data, asynchronous communication, algorithmic triage, and interoperability across settings. When data rights, privacy safeguards, clinical accountability, and escalation pathways are poorly specified, trust falls and sustainability becomes unlikely. Governance, therefore, should be treated as an implementation variable rather than a late-stage policy add-on.

## Assessing value beyond adoption

The current literature often uses a narrow definition of success. Many studies still emphasize uptake, engagement, satisfaction, or short-term behavior change, but health systems must decide whether a DHT improves care enough to justify ongoing investment. That decision requires a broader concept of value.

Recent value frameworks are helpful because they move beyond simplistic questions such as “does it work?” A Delphi-derived framework for patient-facing DHTs identifies value domains spanning health inequalities, data rights and governance, technical and security characteristics, economic characteristics, clinical characteristics, and user preferences (7). A related framework for provider-facing DHTs shows that stakeholders also judge value through workflow consequences, information quality, implementation burden, and governance considerations (8). These multidimensional approaches are especially relevant for chronic disease management, where an intervention may improve continuity or self-management support without immediately changing a biomarker, or may improve outcomes for one group while excluding another.

Seen through this lens, some current evidence becomes easier to interpret. For example, a CKD intervention may generate high activation and favorable usability while revealing that certain features carry more patient value than others (10). A telehealth model may expand geographic reach while still failing on affordability, workload, or equity. A care-grade evaluation should therefore capture at least four domains: clinical outcomes, service outcomes, patient and clinician experience, and equity-governance consequences.

## A care-grade roadmap for digital chronic care

The literature reviewed above suggests that digital chronic care should move from isolated pilots to accountable service platforms. Five shifts are especially important.

First, co-design should be operational rather than symbolic. Stakeholder mapping and structured co-design methods can clarify who uses the system, who carries new work, and which trade-offs matter most in specific contexts (21, 22). Second, implementation science should be treated as a routine quality requirement. Studies that ignore implementation strategies, contextual barriers, and adaptation logic leave decision-makers with little guidance for replication or scale (3, 11, 12).

Third, equity should be designed in from the start. Low-bandwidth pathways, digital navigation support, literacy-sensitive content, and locally relevant onboarding are essential in rural, low-resource, and otherwise marginalized settings (9, 13, 19, 20). Fourth, evaluation should include value domains that matter to health systems, not just metrics that are easy to collect. This means measuring clinician workload, continuity, responsiveness, safety, implementation cost, and stratified outcomes alongside traditional clinical endpoints (7, 8, 11). Fifth, DHTs should be embedded within learning systems that use routine data to refine workflows, personalize support, and improve care over time (23, 24).

Operational implications. In practical terms, pilot implementations should pre-specify measures across (a) clinical endpoints such as disease control, exacerbations, and hospitalizations; (b) service outcomes such as continuity, response times, escalation reliability, and clinician workload; (c) patient-reported outcomes and experience; (d) equity indicators stratified by geography, connectivity, age, language, affordability, and digital literacy; and (e) economic outcomes including patient costs, program costs, and cost-effectiveness. Mixed-methods evaluation should document not only whether a DHT performs, but also how workflows were redesigned, what implementation strategies were used, what governance safeguards were required, and why performance varied across settings (see Table 2).

**Table 2:** Common evidence and implementation gaps, and practical responses for revision of digital chronic care research and rollout.

Persistent gap	How it appears in the literature	Why it matters	Practical response
Unclear scope and evidence logic	Cross-condition examples are presented without stating why those studies were selected.	Weakens coherence and makes synthesis seem opportunistic.	State the article as a perspective and define the analytic questions guiding evidence selection.
Over-reliance on early implementation outcomes	Many studies stop at acceptability, feasibility, or adoption (3, 11).	Acceptable tools may still be clinically ineffective, inequitable, or unsustainable.	Pair implementation outcomes with service, clinical, economic, and equity outcomes.
Workflow fit assessed too late	Patient and clinician burden becomes visible only after rollout (5, 6).	Poor fit drives abandonment even when users see theoretical benefit.	Map tasks, accountability, escalation pathways, and data review responsibilities during design.
Equity treated as a secondary issue	Digital divide and literacy barriers are reported after scale-up problems emerge (9, 13, 19, 20).	Risks intervention-generated inequalities and misleading claims of access improvement.	Design low-bandwidth, affordable, literacy-sensitive, and locally supported models from the outset.
Value defined too narrowly	Benefit is inferred from engagement or satisfaction alone (7, 8, 10).	Health systems cannot judge readiness for sustained adoption or reimbursement.	Use multidomain value frameworks spanning outcomes, experience, workflow, economics, and governance.
Insufficient attention to adaptation and sustainability	Studies rarely explain how interventions change over time or across organizations (12).	Scale-up becomes trial-and-error and lessons are not transferable.	Report implementation strategies, context, adaptations, and sustainment plans transparently.

## Discussion

Digital health for chronic disease management has progressed beyond novelty, but the field still overproduces tools and underproduces durable integration. The evidence reviewed here suggests that the central bottleneck is not whether digital tools can be built, but whether they are embedded within care models that patients, clinicians, and organizations can realistically sustain. This requires a shift from tool-centric evaluation to system-aware evaluation that recognizes workflow redesign, equity, and governance as core determinants of performance.

This perspective also highlights why reviewer concerns about coherence, definitions, and evidence framing matter substantively rather than stylistically. When key terms such as integration, value, or adoption remain vague, the literature can appear more consistent than it truly is. Clarifying these concepts shows that digital chronic care succeeds only when technical functionality, implementation quality, equity, and governance are aligned.

*Limitations and evidence needs*: This article synthesizes published evidence and proposes a conceptual roadmap rather than presenting new empirical data. The breadth of chronic disease contexts and technologies discussed limits condition-specific detail, and the evidence cited was selected purposively rather than through a systematic review protocol. Accordingly, the recommendations advanced here should be interpreted as an integrated argument grounded in the literature, not as a formal comparative effectiveness conclusion. Future work should test these propositions through pragmatic trials, hybrid effectiveness-implementation studies, and real-world evaluations with transparent reporting of context, adaptation, and differential effects across population groups.

In conclusion, digital chronic care will create reliable value only when technologies are treated as components of accountable service redesign. Care-grade integration requires more than adoption: it requires fit with workflows, explicit implementation logic, equity-sensitive delivery, multidimensional value assessment, and governance that patients and clinicians can trust. Under those conditions, digital health can move from scattered pilots to sustainable platforms for chronic disease management.

## Data Availability

The original contributions presented in the study are included in the article/supplementary material, further inquiries can be directed to the corresponding author.
